# Comparing low volume of blood flow restricted to high-intensity resistance training of the finger flexors to maintain climbing-specific strength and endurance: a crossover study

**DOI:** 10.3389/fspor.2023.1256136

**Published:** 2023-09-29

**Authors:** Tomáš Javorský, Atle Hole Saeterbakken, Vidar Andersen, Jiří Baláš

**Affiliations:** ^1^ Faculty of Physical Education and Sport, Charles University, Prague, Czech Republic; ^2^ Department of Sport Science, University of Innsbruck, Innsbruck, Austria; ^3^ Faculty of Education, Arts and Sports, Western Norway University of Applied Sciences, Sogndal, Norway

**Keywords:** injury, hypertrophy, hypoxia, ischemia, intermittent exercise, isometric contraction, strength, oxidative capacity

## Abstract

**Introduction:**

It is acknowledged that training during recovery periods after injury involves reducing both volume and intensity, often resulting in losses of sport-specific fitness. Therefore, this study aimed to compare the effects of high-intensity training (HIT) and low-intensity training with blood flow restriction (LIT + BFR) of the finger flexors in order to preserve climbing-specific strength and endurance.

**Methods:**

In a crossover design, thirteen intermediate climbers completed two 5-week periods of isometric finger flexors training on a hangboard. The trainings consisted of ten LIT + BFR (30% of max) or HIT sessions (60% of max without BFR) and were undertaken in a randomized order. The training session consisted of 6 unilateral sets of 1 min intermittent hanging at a 7:3 work relief ratio for both hands. Maximal voluntary contraction (MVC), force impulse from the 4 min all out test (W), critical force (CF) and force impulse above the critical force (W') of the finger flexors were assessed before, after the first, and after the second training period, using a climbing-specific dynamometer. Forearm muscle oxidative capacity was estimated from an occlusion test using near-infrared spectroscopy at the same time points.

**Results:**

Both training methods led to maintaining strength and endurance indicators, however, no interaction (*P* > 0.05) was found between the training methods for any strength or endurance variable. A significant increase (*P *= 0.002) was found for W, primarily driven by the HIT group (pretest—25078 ± 7584 N.s, post-test—27327 ± 8051 N.s, *P *= 0.012, Cohen's *d* = 0.29). There were no significant (*P* > 0.05) pre- post-test changes for MVC (HIT: Cohen's *d* = 0.13; LIT + BFR: Cohen's *d* = −0.10), CF (HIT: Cohen's *d* = 0.36; LIT + BFR = 0.05), W` (HIT: Cohen's *d* = −0.03, LIT + BFR = 0.12), and forearm muscle oxidative capacity (HIT: Cohen's *d* = −0.23; LIT + BFR: Cohen's *d* = −0.07).

**Conclusions:**

Low volume of BFR and HIT led to similar results, maintaining climbing-specific strength and endurance in lower grade and intermediate climbers. It appears that using BFR training may be an alternative approach after finger injury as low mechanical impact occurs during training.

## Introduction

Sport climbers heavily rely on finger flexor contractions, making finger flexor strength and endurance crucial predictors of climbing performance (1, 2). Previous research has extensively investigated the physiological adaptations induced by high-intensity training (HIT) on finger strength and endurance (3, 4). For example, specific maximal strength and hypertrophy training designed for climbers have demonstrated significant increases in finger flexor strength and endurance after 5–10 weeks of training (5–8). However, HIT of the finger flexors may increase the risk of injuries in the fingers, hands, elbows, or shoulders, with chronic injuries being the most common among sport climbers (9, 10). Moreover, when recovering from injuries such as pulley ruptures or strains it is recommended to gradually increase training loads (11). Consequently, recovery periods require climbers to train with decreased intensity, often resulting in losses of sport-specific fitness.

An alternative approach to HIT for improving or maintaining finger strength and muscle hypertrophy is training at low intensities (typically 20%–40% of maximum strength) with blood flow restriction (LIT + BFR), achieved by applying external pressure to the limb proximal to the working muscle (12). LIT + BFR exercise creates a localized hypoxic environment and promotes recruitment of both types I and II muscle fibres, leading to enhanced muscle strength and power (13–15). Furthermore, changes in key markers of protein synthesis, such as mTOR and HIF-1, support the observed adaptations in the muscle following LIT + BFR training (16, 17). Accordingly, LIT + BFR triggers an upregulation of protein synthesis, facilitating muscle growth and strength gains despite the use of lower training loads (decreased mechanical stress). This suggests that the metabolic stress induced by LIT + BFR exercise can stimulate muscle protein synthesis to a comparable extent as high-intensity exercise (18, 19). To date there are no studies comparing HIT and LIT + BFR in climbing-specific hangboard resistance training. However, based on the existing literature, it is reasonable to hypothesize that LIT + BFR and HIT may yield comparable effects in finger flexors training in climbers.

Previous research has shown that increasing strength can be achieved with low volume of HIT per week (20, 21). However, it remains unknown whether the same training volume of LIT + BFR would yield similar effects. Most studies investigating blood flow restriction (BFR) interventions have primarily focused on designs maximizing their effectiveness for increasing muscle strength and hypertrophy (22, 23). However, during the recovery period following an injury, the primary objective of training is to maintain strength and endurance levels using minimal load and training volume (20). Low-intensity training (LIT) with BFR training has been proposed and utilized as a method of recovery after various types of injuries in lower limbs such as knee osteoarthritis (24) or arthroplasty (25), however, to authors best knowledge, there is not any literature available on this topic on the upper extremities related to the climbing.

Therefore, the objective of this study was to investigate the effects of low volume of LIT + BFR training and HIT on maintaining climbing-specific strength and endurance. We hypothesised that HIT and LIT + BFR will be equally effective in preserving sport specific strength and endurance in intermediate climbers.

## Methods

### Participants

Thirteen lower grade to intermediate climbers [6 male, 7 female participants: males—age, 24.3 ± 2.0 yrs; climbing ability level 13 ± 4 IRCRA (International Rock Climbing Research Association) grade; females—age, 32.6 ± 12.5 yrs; climbing ability 9 ± 2 IRCRA grade] volunteered to take part in the study. Participants self-reported their climbing ability using French/Sport grade which was transformed to the IRCRA difficulty scale ranging from 1 to 32 (26). At the beginning, all participants completed written informed consent forms and medical health questionnaires. Exclusion criteria included venous thrombosis, cardiovascular diseases (including high blood pressure and diabetes), unexplained chest pain, heart pathologies, and fainting during physical activities. Additionally, participants with carpal tunnel syndrome, acute upper limb injuries, tendosynovitis, or tendon injuries in the upper limb, pregnancy, or in the injury recovery phase were also excluded.

Participants were instructed to abstain from engaging in any strenuous exercise, consuming caffeine, and consuming alcohol within 24 h before each experimental testing session. Furthermore, participants were not allowed to maintain normal training routine or engage in any finger flexor strength and endurance training. This was achieved partially by the ongoing COVID lockdown when sport facilities were closed. Additionally, participants were asked to continue their regular dietary and supplement habits. The study was approved by Ethics Committee of Charles University, Faculty of Physical Education and Sport. The participants provided their written informed consent to participate in this study.

### Experimental protocol

**Figure 1:**
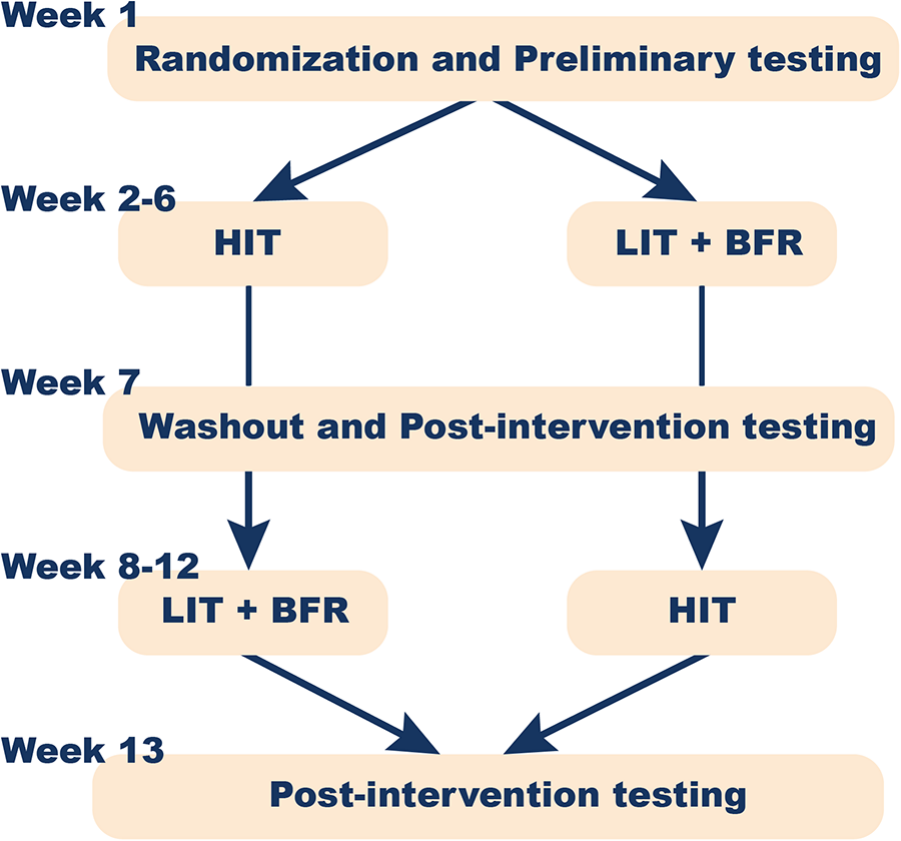
Experimental design of the study.

The 13 weeks long experimental protocol is depicted at Figure 1. All participants completed two 5 weeks periods of finger flexors training in a cross-over randomized order with a 1-week long washout period. The two training interventions consisted of either isometric HIT or LIT + BFR on a hangboard. Testing climbing specific strength and endurance was applied before and after each period of training (Figure 1).

To eliminate interference between individual tests, the participants underwent two separate testing sessions during the testing week. In the first session, the muscle oxidative capacity and the maximal voluntary contraction (MVC) were assessed. The second testing session involved performing a 4-min all-out test after the measurement of blood pressure to determine the level of occlusion.

Upon their first visit, participants were randomly assigned into two groups based on the training intervention. They were also familiarized with the laboratory setup. Additionally, they completed a questionnaire and signed the medical consent form. In the questionnaire, participants reported their climbing ability as proposed by Draper et al. (26).

### Warm-up

All subjects completed a standardized self-directed warm-up prior to the assessment and training protocol. The warm-up consisted of three minutes of pulse-raising activity, such as jogging or cycling, followed by three minutes of climbing, which is considered a sport-specific activity. In addition, the warm-up included a series of 5:5 s work-to-rest ratio hangs on the testing edge in a half-crimp position at ∼50% of the perceived maximum force (27).

### Training interventions

Both training interventions consisted of 10 training sessions (2 sessions per week during each 5-week period). The LIT + BFR and HIT participants previously scheduled a time of the day for the individual sessions of hangboard strength exercises. The intensity for each training type was based on the MVC tested prior to each intervention. The training was performed on the same wooden rung as for testing MVC and all-out test (see below) in standing position with arms ∼180° flexed in shoulder, and slightly flexed in elbows. Participants applied the target force on the rung by hanging (bending the knees). The online feedback of applied force was visible on the screen of the testing/training device (1D-SAC, Spacelab, Sofia, Bulgaria).

#### Blood flow restriction training

**Figure 2:**
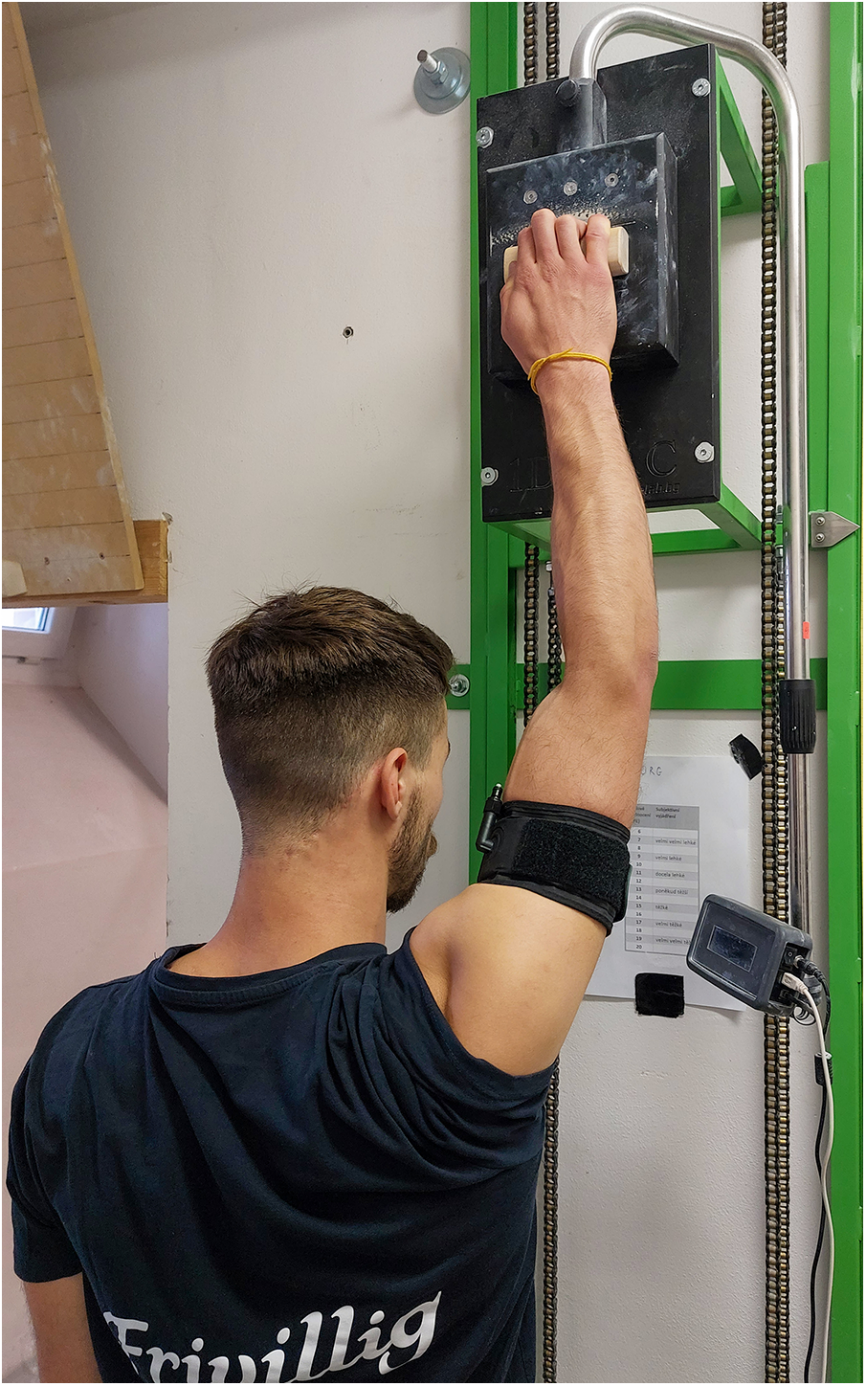
Position of participant during the Low-intensity training with the blood flow restriction.

To implement BFR, we utilized a cuff provided by Occlude ApS (Aarhaus, Denmark). Prior to each training session, the cuff was inflated to 60% of the complete arterial occlusion pressure (21, 28) on training arm, which caused decrease in the blood flow in the downstream vascular system by 47%–48% (29). In each session both arms performed 6 sets over two blocks (one block consisted of three consecutive sets) unilaterally for each arm, and each set comprising 6 repetitions performed at 30% of MVC, with a work-to-rest ratio of 7 to 3 s. Following the completion of set 3 (60 s rest in between) for one arm, the cuff was deflated and participants immediately continued with the other arm for next three sets. In total, 36 isometric contractions for each arm were completed (Figure 2). The cuff pressure was monitored and controlled during the rest periods between sets.

#### High-Intensity training

Participants performed HIT sessions at 60% of their MVC. The same volume of training as for LIT + BFR was applied. Each training session consisted of 12 working sets (i.e., 6 sets of each arm divided into two blocks with 5 min rest in between), with each set comprising 6 repetitions and a work-to-rest ratio of 7 to 3 s. Following the completion of the third set, participants were given a 5 min recovery period while the other arm was exercising.

### Testing climbing specific strength and endurance

#### Maximal strength

The maximal strength of the finger flexors was determined using a custom-made dynamometer (1D-SAC, Spacelab, Sofia, Bulgaria). The participant was instructed to maintain a 5 s long half-crimp position while “hanging” on the wooden rung. The rung depth was 23 mm with a 10 mm radius to maximize the activation of the flexor digitorum profundus (FDP) and flexor digitorum superficialis (FDS) (30). Two attempts were performed separated by a two-minute rest in between. Participants were instructed to progressively transfer as much of their weight as possible onto the wooden rung with their dominant arm. The highest peak value from the two trials was considered as the MVC of finger flexors, and this value was used to determine relative workloads for the following training intervention.

#### All-out test

To assess the critical force (CF), force impulse from all contractions (W), and impulse above the critical force (W'), the 4-min all-out test was performed (31). This test involved 24 isometric maximal voluntary contractions on the same rung as for maximal strength (1D-SAC, Spacelab, Sofia, Bulgaria) in a half crimp position with a 7:3 s work to rest ratio.

**Figure 3:**
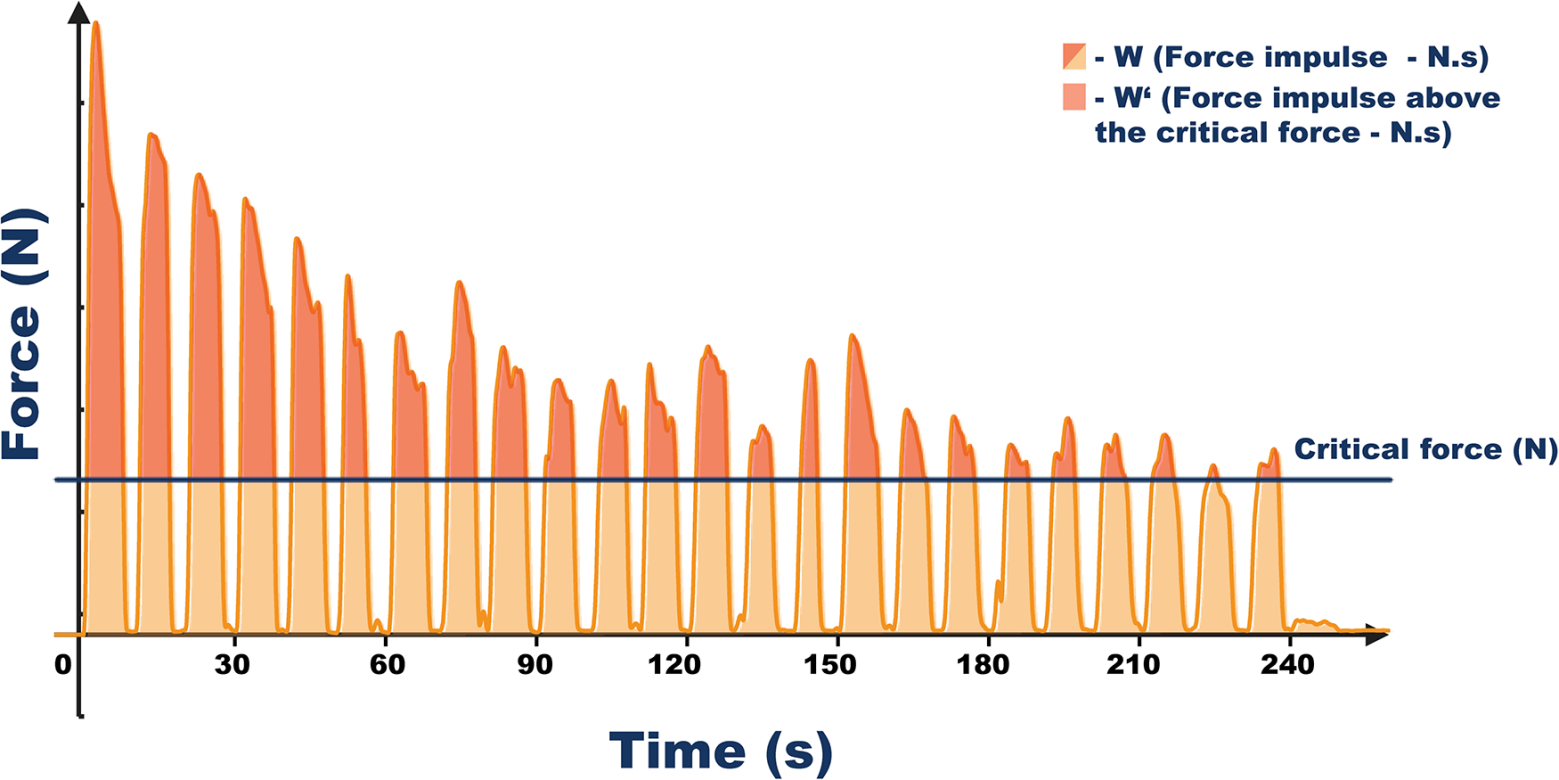
Vizualization of data acquired by the all-out test for the finger flexors. Critical force was calculated as the average force from the last three contractions. The duration of the all-out test was 240 s with 7:3 work to rest ratio. Force impulse from all contractions was calculated as the area under the force-time curve and represents total isometric muscle work during the test (**W**). Impulse above the critical force represents energy store component (W’).

During the “rest” phase, participants were instructed to maintain the anatomical position with upper-limb over the head level and were not allowed to shake their forearms or hands, as shaking is known to aid recovery (32). However, participants could dry their fingers using the chalk. Loud verbal encouragements were given to all participants to reach their maximum force during every contraction. Force and time data were continuously recorded throughout the test. For the visual representation see Figure 3.

For each contraction in all tests, the length (in seconds), peak and mean force (in kilograms), and the impulse were determined. The CF was defined as the mean force from the last three contractions of the test.

#### Muscle oxidative capacity

To assess the muscle oxidative capacity, near-infrared spectroscopy (NIRS) (Portamon, Artinis Medical Systems BV, The Netherlands) was employed to monitor changes in tissue oxygenation levels of the FDP. A chartered physiotherapist located the FDP using the technique recommended by Schweizer and Hudek (30), where the thumb and first finger were squeezed together, and the middle of the muscle belly was palpated (30). The NIRS device sampling frequency was set to 10 Hz and data were processed using the Oxysoft software (Artinis Medical System, BV, The Netherlands). Path length factor was set to 4. Muscle oxidative capacity was estimated by calculating half-time to recovery of the tissue oxygen saturation (O_2_HTR) after arterial occlusion (33).

Participants were instructed to rest in a supine position with their arm elevated above heart level for 20 min after fitting the artery tourniquet. Following the initial measurement of the baseline, the tourniquet was inflated to a supramaximal pressure of 250 mmHg for 5 min. After that, the cuff was rapidly released, and recovery muscle tissue oxygen saturation (StO_2_) values were recorded for 3 min. Half-time of StO_2_ recovery was calculated, which represents a valid estimate of oxidative capacity (33).

### Statistical analysis

Statistical analyses were performed using IBM SPSS for Windows (IBM Corp. Released 2020. IBM SPSS Statistics for Windows, Version 27.0. Armonk, NY: IBM Corp). Descriptive statistics (mean ± standard deviation) were used to characterize strength and endurance indicators during pretest and post-test. The analysis of variance (ANOVA) 2 × 2 with repeated measures was conducted to examine the main effects of time (pretest vs. post-test) and training method (LIT + BFR vs. HIT), as well as their interaction effect. The significance level was set at *P *< 0.05. Post hoc analysis using Bonferroni correction was performed to compare specific pairs of interventions in terms of their effects on the pretest and post-test measures. Effect sizes of 0.3, 0.5, and 0.8 were interpreted as small, medium, and large effects, respectively (34). Utilizing the Shapiro-Wilk test, all data were determined to be normal and met the criteria of Mauchly's test of sphericity.

## Results

At baseline, no differences for were observed between the training methods for any of the variables (*P* > 0.05).

There was a significant main effect of time for impulse (delta W =  + 1568 Ns; *P *= 0.002). However, there was no significant interaction of time and training method demonstrating no substantial differences between LIT + BFR and HIT (*P = *0.057–0.855).

**Figure 4:**
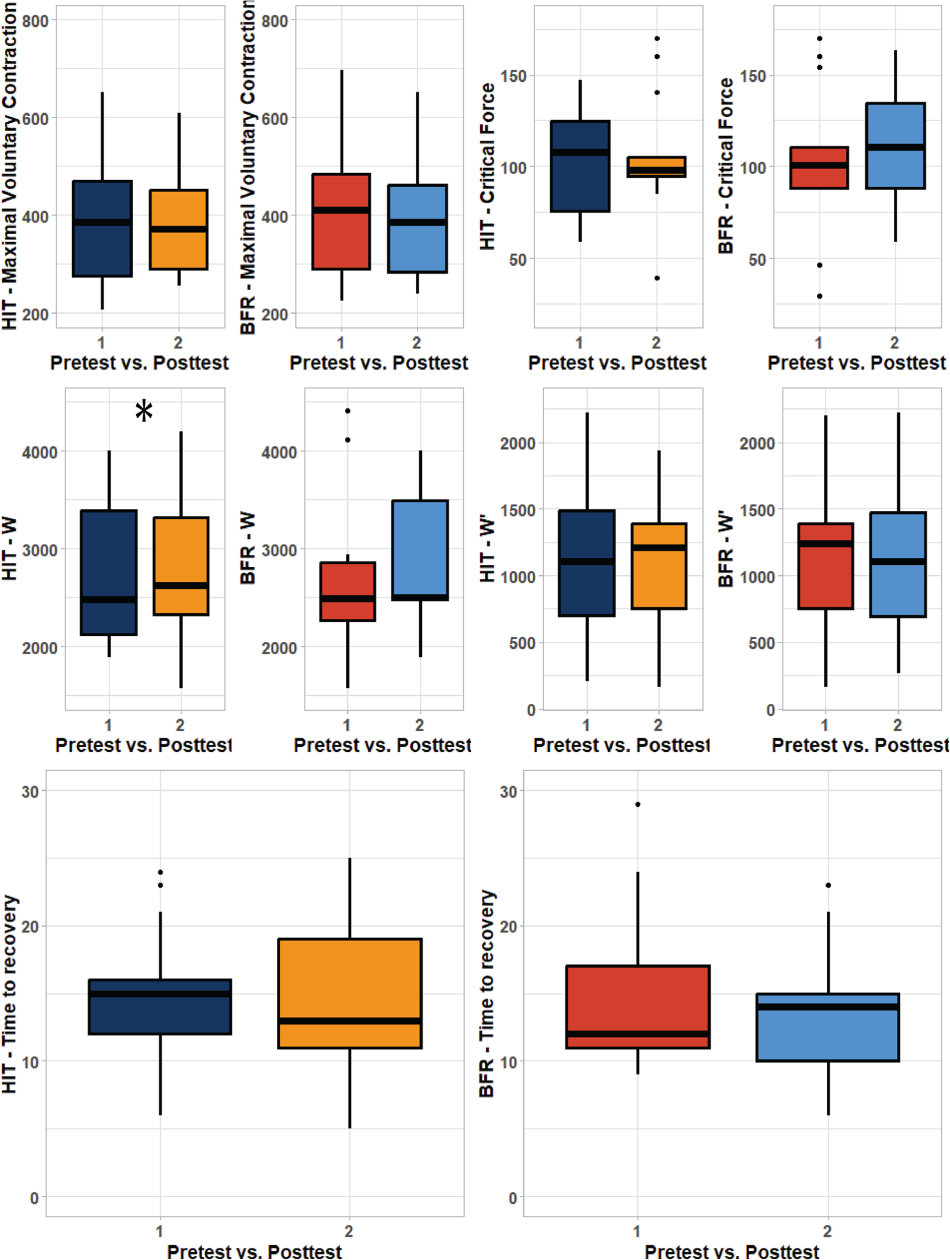
Boxplot visualization of pretest post-test results. Left panel represent high intensity training (HIT) while right panels represent low intensity training with blood flow restriction (LIT + BFR) The area of box shows quartile and whiskers represent 1.5 interquartile range between the first and third quartile. The line in the middle corresponds to the mean value. W—impulse, W’—impulse above the critical force, O_2_HTR—oxygen ½ time to recovery after occlusion. * represents significant improvements from pretest (*P *< 0.05).

Pairwise comparisons showed significant increases of force impulse only for HIT method (Table 1, Figure 4). Otherwise, non-significant improvements with small or no effect size were found for all strength and endurance indicators and no significant decreases of climbing specific strength or endurance indicators were demonstrated (Table 1, Figure 4).

**Table 1:** Mean (± standard deviation) score of pretest and post-test measurements for high intensity training (HIT) and low intensity training with blood flow restrictions (LIT + BFR).

	HIT				LIT + BFR			
	Pretest	Post-test	*P*	Cohen's *d*	Pretest	Post-test	*P*	Cohen's *d*
MVC (N)	356 ± 134	373 ± 113	0.241	0.13	376 ± 138	362 ± 125	0.158	−0.10
Cf (N)	103 ± 26	113 ± 30	0.237	0.36	114.3 ± 31	116 ± 30	0.844	0.05
W (N.s)	25,078 ± 7,583	27,327 ± 8,051	0.012	0.29	26,661 ± 8,415	27,551 ± 6,593	0.392	0.12
W’ (N.s)	10,246 ± 6,011	10,092 ± 5,979	0.845	−0.03	9,494 ± 5,278	10,152 ± 5,599	0.353	0.12
O_2_HTR (s)	14.3 ± 5.1	13.1 ± 5.1	0.569	−0.23	13.6 ± 4.9	13.2 ± 4.8	0.830	−0.07

W, impulse from the 4 min all-out test; W’, impulse above the critical force; CF, critical force; O_2_HTR, oxygen saturation ½ time to recovery after arterial occlusion.

## Discussion

The main finding of the current study was that small volume of LIT + BFR was equally effective as HIT to maintain finger flexor strength and endurance in lower grade and intermediate climbers.

To evaluate maximum finger flexor strength, we employed an ecological setting with the arm positioned overhead without any fixation. This method has been demonstrated to be a valid and reliable measure of climbing-specific strength, with a standard error of measurement (SEM) of 35 N (35). Neither the HIT, nor LIT + BFR interventions resulted in significant changes in finger flexor strength. The observed pretest-post-test changes fell within the previously mentioned SEM range. It has been observed that strength decreases occur rapidly with a training interruption, becoming more pronounced after 8 days of inactivity (36) It is hypothesized that neural factors such as motor unit recruitment and synchronization, firing frequency, and intramuscular coordination are responsible for strength losses during the early stages of inactivity, while morphological factors contribute to greater strength decreases thereafter (37). Our study demonstrates that low volume of intermittent isometric HIT (60% MVC, with a total exercise time of 36 × 10:3 s work: relief cycles per session, two sessions per week) and an equivalent volume of low-intensity with BFR (30% MVC) were effective in maintaining the initial strength level for 5 weeks. All participants were able to sustain both training protocols without premature localized exhaustion. Therefore, it may be speculated that 2 sessions per week, with a total of 12 min of isometric non-exhaustive exercise per arm at low intensity and with BFR, counteracted the deteriorating changes that neural factors may have on maximal strength due to inactivity.

During high-intensity resistance training, a single set of 6–12 repetitions with loads ranging from approximately 70%–85% 1 repetition maximum 2–3 times per week reaching volitional or momentary failure for 8–12 weeks can produce suboptimal, yet significant increases in squat and bench press strength in resistance-trained men (20). Our non-exhaustive protocol with smaller muscle groups, slightly lower intensity, and similar volume did not result in significant improvements. It appears that exhaustive protocols are necessary to induce structural changes leading to strength increases (38, 39). However, a similar volume of non-exhaustive exercise may have benefits in maintaining the current level of strength.

LIT + BFR training does not only have impact on maximal strength improvements but may also, due to peripheric and central adaptations, have direct or indirect impact on endurance performance (40, 41). In our study, we estimated endurance of the finger flexors using several indicators: W, W', CF from 4 min all out test and O_2_HTR from arterial occlusion test. W is an indicator of total working capacity and represents an overall measure of finger strength and endurance. W’ is the capacity to release energy above the CF and is often related to strength-endurance capacity while the level of CF represents the amount of energy predominantly released by aerobic metabolism (42). O_2_HTR is a standardized NIRS derived functional index estimating muscle aerobic capacity. Faster recovery of FDP has been associated with increased climbing ability (43). Similar to maximal strength, no decreases in any endurance indicators were observed. On the contrary, after HIT, W was statistically higher, suggesting that low volume of HIT may lead to overall improvement in finger flexor working capacity in intermediate climbers as W represents both strength and endurance components. However, the effect size for improvement changes was low, and no differences between the two methods were found. The maintenance of all endurance indicators during 5-weeks LIT + BFR training is very promising as submaximal resistance to fatigue appears to be deteriorated to a greater extent from training interruption in comparison with maximal force and maximal power (37).

Endurance adaptations following LIT + BFR training have been associated with improvements in macro- and microvascular functions, muscle redox and ionic buffering, and mitochondrial respiratory capacity (40, 41). In our study, the aerobic capacity of the finger flexor muscles was estimated from the NIRS signal. It is important to note that the sensitivity of StO_2_ recovery as a training indicator in climbers is still unknown, and further experimental studies are needed to validate its use. Subsequent studies should also aim to investigate the pathways explaining forearm oxidative capacity and consider using NIRS technology to independently assess skeletal muscle oxygen diffusion capacity and mitochondrial respiratory capacity (44).

There are other strength and limitations to be stated. A strength of the study is that all participants refrained from engaging in any climbing-specific or upper-body strength activities during the 13-week experimental period, ensuring that any observed changes could be attributed to our experimental conditions. The intervention may be regarded as a simulation of a rehabilitation period. Participants were fit enough to train under controlled environment but could not train/climb in an uncontrolled environment due to lock-down restrictions. The crossover design allowed for a direct comparison between the two training modalities within the same group of participants, minimizing inter-individual variability (45). However, due to time requirements, a relatively short one-week washout period between the training interventions was applied. Of note, a control group was not included which might be useful of quantifying no strength training or the short washout period. Nevertheless, this does not seem to influence our results as no changes in any indicator were observed after the HIT or LIT + BFR intervention. The small group size in this study may limit the generalizability of the findings and the ability to detect small differences between the training modalities. Moreover, using BFR with more advanced climbers may have provided different results. MVC was assessed only once before each training intervention to set the training load. In other words, the climbers trained at the same relative intensity throughout the whole period. This may also explain the lack of changes during the different periods. If MVC was tested every week, there may had been a progression in the training which ultimately may have led to an increase in (some of) the variables. On the other hand, during recovery periods from an injury, regular testing of MVC would increase stress on injured tissues and may slow the recovery process.

Our findings support the hypothesis that both approaches, with and without BFR, were equally effective in preserving the studied parameters during the minimal training period. However, it is important to note that physiology of these adaptations may differ during exercise at 30% of MVC compared to higher intensity exercise (23, 46, 47). Therefore, BFR training at a lower intensity (30% of MVC) appears to be a viable substitute for HIT during recovery periods and may offer advantages, particularly for climbers recovering from injuries, although it is more discomforting and less enjoyable compared to HIT (48).

In conclusion, this study demonstrates that low volume of non-exhaustive BFR training at a lower intensity can be as effective as HIT in preserving sport-specific strength and endurance. These findings suggest that LIT + BFR training may be a viable alternative for climbers recovering from injuries.

## Data Availability

The original contributions presented in the study are included in the article/**Supplementary Material**, further inquiries can be directed to the corresponding author.
